# Transcriptomic Analyses Reveal Novel Genes with Sexually Dimorphic Expression in the Zebrafish Gonad and Brain

**DOI:** 10.1371/journal.pone.0001791

**Published:** 2008-03-12

**Authors:** Rajini Sreenivasan, Minnie Cai, Richard Bartfai, Xingang Wang, Alan Christoffels, Laszlo Orban

**Affiliations:** 1 Reproductive Genomics Group, Temasek Life Sciences Laboratory, Singapore, Singapore; 2 Department of Biological Sciences, National University of Singapore, Singapore, Singapore; 3 Computational Biology, Temasek Life Sciences Laboratory, Singapore, Singapore; 4 School of Biological Sciences, Nanyang Technological University, Singapore, Singapore; University College Dublin, Ireland

## Abstract

**Background:**

Our knowledge on zebrafish reproduction is very limited. We generated a gonad-derived cDNA microarray from zebrafish and used it to analyze large-scale gene expression profiles in adult gonads and other organs.

**Methodology/Principal Findings:**

We have identified 116638 gonad-derived zebrafish expressed sequence tags (ESTs), 21% of which were isolated in our lab. Following *in silico* normalization, we constructed a gonad-derived microarray comprising 6370 unique, full-length cDNAs from differentiating and adult gonads. Labeled targets from adult gonad, brain, kidney and ‘rest-of-body’ from both sexes were hybridized onto the microarray. Our analyses revealed 1366, 881 and 656 differentially expressed transcripts (34.7% novel) that showed highest expression in ovary, testis and both gonads respectively. Hierarchical clustering showed correlation of the two gonadal transcriptomes and their similarities to those of the brains. In addition, we have identified 276 genes showing sexually dimorphic expression both between the brains and between the gonads. By *in situ* hybridization, we showed that the gonadal transcripts with the strongest array signal intensities were germline-expressed. We found that five members of the GTP-binding *septin* gene family, from which only one member (*septin 4*) has previously been implicated in reproduction in mice, were all strongly expressed in the gonads.

**Conclusions/Significance:**

We have generated a gonad-derived zebrafish cDNA microarray and demonstrated its usefulness in identifying genes with sexually dimorphic co-expression in both the gonads and the brains. We have also provided the first evidence of large-scale differential gene expression between female and male brains of a teleost. Our microarray would be useful for studying gonad development, differentiation and function not only in zebrafish but also in related teleosts via cross-species hybridizations. Since several genes have been shown to play similar roles in gonadogenesis in zebrafish and other vertebrates, our array may even provide information on genetic disorders affecting gonadal phenotypes and fertility in mammals.

## Introduction

Zebrafish (*Danio rerio*) has become a major vertebrate model for developmental biology and genetics [1], genomics [2], [3] and human diseases [4]–[6] during the last three decades. Despite these advances, little is known about the molecular aspects of its reproduction. The molecular mechanisms underlying gonad development and function in zebrafish have yet to be elucidated.

Gonad differentiation in zebrafish is a highly complex process involving a ‘juvenile ovary-to-testis’ transformation [7]–[9]. At 15–20 days post fertilization (dpf), every zebrafish individual develops an ovary-like gonad (‘juvenile ovary’). The juvenile ovary continues to develop until maturation in the females while in the rest of the individuals it undergoes a transitional intersexual phase eventually leading to the formation of a differentiated testis [7], [10]. This process is further complicated by the high variability of the starting point and extent of the transformation process in different individuals [9]. Although the molecular mechanism of gonad differentiation is largely unknown, the products of several genes, including aromatase (*cyp19a1*; [11], Fushi tarazu factor-1d (*ff1d*; [12], [13]), anti-Müllerian hormone (*amh*; [14], [15]), and 11β-hydroxylase (*cyp11b*; [15]) have been implicated in this process.

Besides the gonads, the brain is also involved in vertebrate reproduction. The involvement of the brain in gonad development has been established by studies on quail which showed that males transplanted with female forebrains had abnormally developed testes, indicating that a genetically male brain is necessary for normal testis development [16]. Since sex change in some teleosts can be influenced by social control [17], [18], it is likely that events in the brain may determine the fate of the gonads, as suggested earlier by Francis [19]. Vertebrate reproduction and in part, sexual development, is largely controlled by feedback interactions in the brain-pituitary-gonadal (BPG) axis [19], [20]. Contrary to the dogma in mammals and birds that brain sexual dimorphism is entirely a result of hormonal action from the gonads, it has now been proven that the brain also develops differently in the two sexes as a result of differential brain gene expression, remarkably even before the gonads are formed [21]–[23].

Microarray-based approaches have been used to identify genes involved in gonad development and function in several organisms. In *Caenorhabditis elegans,* sex-regulated genes have been identified by characterizing gene expression differences between males and hermaphrodites using DNA microarrays [24], while in *Drosophila melanogaster*, microarray analysis has been performed to obtain the gene expression profile of the testis [25]. In rainbow trout, precocious ovaries were compared against normal ones using ovary-specific microarrays to reveal genes which are potentially involved in ovary maturation and development [26]. Extensive array-based studies have also been performed to elucidate gonad development and function in mice. These include gene expression profiling in differentiating male and female embryonic gonads [27], microarray analyses to identify genes involved in ovary development [28] and time course profiling of testicular gene expression during spermatogenesis [29].

In contrast to the extensive studies performed on other organisms, there has been little information available regarding the gonad transcriptomes of zebrafish. Currently available zebrafish microarrays include at least seven commercial whole genome oligo arrays, a heart and skeletal muscle-derived cDNA array [30], a microarray derived from mixed tissues of 4-month-old to adult zebrafish [31], an array containing brain-specific cDNAs [32] and a microarray spanning promoter regions of over 11,000 genes [33].

In order to identify and characterize genes potentially involved in gonad development and function, we constructed a microarray comprising nearly 6,400 unique cDNAs from zebrafish gonads. In our previous work, we analyzed a smaller subset of this collection by hybridizing adult ovary-, testis- and kidney-derived labeled targets to a membrane-based cDNA macroarray containing 2760 adult gonad cDNAs [34]. In the current experiment, we extended the throughput of the study by adding 3634 clones (derived mostly from the differentiating gonad) to the array and switching to a slide-based platform. In order to analyze the transcriptomes of the adult gonads and to compare them with those of other organs, RNA from adult ovary and testis were used as targets for hybridization onto the microarray, while RNA from the brain, kidney and rest of the body from both male and female adult zebrafish were used as controls. Our analysis revealed that a large number of genes showed sexually dimorphic expression in the gonads and other organs of adult zebrafish. These included novel transcripts which have not been characterized in any other vertebrate species according to our knowledge.

## Results and Discussion

### Extension of the gonadal EST set and generation of the Gonad Uniclone Microarray

We generated 4613 new ESTs from the adult zebrafish testis and ovary and 11010 sequences from the differentiating gonad [3, 4 and 5 weeks post-fertilization (wpf)], which has never been sampled before by others (refer to Table S1 for details). In total, 15623 new zebrafish ESTs have been submitted to GenBank from the effort described in this study.

**Figure 1:**
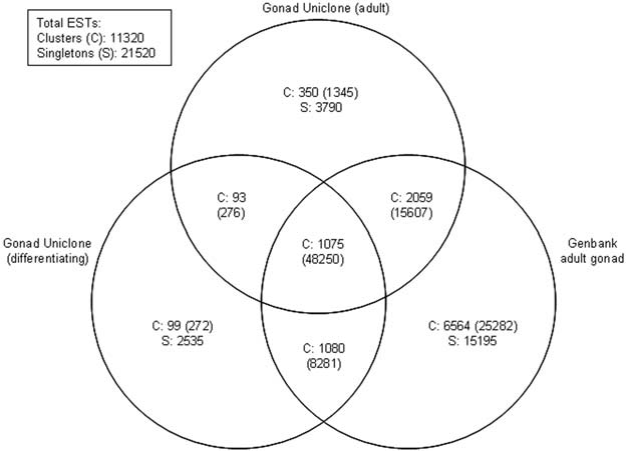
Venn diagram depicting the clones used for *in silico* normalization for the generation of the Gonad Uniclone Microarray. The number of adult and differentiating gonad-derived zebrafish sequences present in our Gonad Uniclone EST collection and their extent of overlap with gonad-derived public zebrafish ESTs listed in GenBank are shown. For details on sequence pre-processing and clustering, refer to the Methods section. In overlapping sections, the total number of clusters, prefaced by ‘C’, is followed in parentheses by the total number of ESTs contained by the clusters. The total number of singletons is prefaced by ‘S’.

In order to minimize redundancy, we performed *in silico* normalization of the above clone set by clustering their sequences together with our clone set published earlier [34], gonad-derived zebrafish ESTs from GenBank and gonadal zebrafish sequences from RefSeq (see Methods for details). Clustering of these sequences resulted in 11320 clusters and 21520 singletons (Fig. 1). Out of these, 542 clusters (representing 1893 ESTs) and 6325 singletons that were isolated by us have not been previously described from the zebrafish gonad by others. When we BLAST-searched our transcripts against GenBank at the nucleotide level (July 2007), 2018 transcripts did not hit any coding regions of the zebrafish genome. Out of these, 90 of them could be translated into peptides, 24 of which contained domains categorized by Gene Ontologies [35] (Table S2). Of the 2018 transcripts, 365 did not find any significant sequence identity to zebrafish ESTs, indicating that they are likely to be absent from commercial zebrafish microarrays. Of the 365 transcripts, 185 did not have hits to any characterized genes in GenBank. Therefore, these sequences represent entirely new zebrafish or vertebrate ESTs, justifying our extension of gene discovery efforts to developmental stages that have not been sampled previously.

From the clustered dataset, we selected full-length cDNA clones representing 6370 unique clusters/singletons to form an extended, *in silico*-normalized cDNA collection, PCR-amplified inserts of which were used to generate our Gonad Uniclone Microarray. All inserts were sequenced from the 5′ end (refer to Table S1 for GenBank IDs) and over 90% of them (5750 clones) from the 3′ end, providing full-length sequences for 3582 clones (GenBank IDs: EX153972-EX159719, EX159734-EX159735).

### The expression profiles of several candidate genes on the Gonad Uniclone Microarray were consistent with previous reports

We performed a total of 32 hybridizations, using eight target organs with four biological replicates each, with the aim of identifying differentially expressed genes in the gonads and other organs, and to set up a biological reference for future hybridizations involving differentiating gonads. The target organs consisted of adult ovary and testis; the brain, kidney and ‘rest-of-body’ (ie. whole body except the three organs listed earlier) from both male and female zebrafish, were used as controls. For simplicity, ‘rest of body’ was regarded as an organ in this publication. All targets were hybridized against a pooled common reference consisting of equal amounts of targets from all dissected organs from a single adult male and a single adult female.

In order to validate our data, duplicate clones of 24 candidate genes, most of which were known to be involved in gonad development and/or function in zebrafish and/or other organisms, were also included in the microarray. We observed a good similarity between expression profiles of the duplicate clones of each candidate gene. Following a 1-way ANOVA test and a Student-Newman-Keuls post hoc test, 18 of the 24 genes showed statistically significant differences between the eight organs. Of the 18 genes, 11 showed at least 1.5-fold higher expression in comparison to the common reference target in either of the gonads. These 11 included four and seven genes most highly expressed in the ovary and testis respectively. Although some of the remaining candidate genes showed enhanced expression in the gonads, they were excluded from analysis for the following reasons: they were expressed more strongly in other organs compared to the gonads, they had inconsistent replicate readings, they fell below the 1.5-fold cutoff or they did not show differential expression between the gonads. In addition, the inability of cDNA microarrays to distinguish between splice isoforms or similar transcripts produced from paralogous loci may also have resulted in lack of the expected differential expression for some of the candidate genes. A summary of the expression pattern observed for each of the 24 candidate genes is described in Table 1 and Table S3. Figure S1 depicts the expression profiles of three selected candidate genes described below.

**Table 1:** The list of the 24 candidate genes on the Gonad Uniclone Microarray, with their expression patterns indicated*.

Genbank ID	Gene name	Gene symbol	Expression pattern
**AY306005**	***17 beta-hydroxysteroid dehydrogenase***	***hsd17b1***	
EF427915	*androgen receptor*	*ar*	
**AY721604**	***anti-Müllerian hormone***	***amh***	**Testis-enhanced**
AF183906	*aromatase a*	*cyp19a1a*	
AF226619	*aromatase b*	*cyp19a1b*	
DQ066429	*desert hedgehog*	*dhh*	
**BC044349**	***estrogen receptor 2a***	***esr2a***	
**BC086848**	***estrogen receptor 2b***	***esr2b***	
**AY249190**	***feminization 1 homolog c***	***fem1c***	**Ovary-enhanced**
BC116585	*forkhead box transcription factor L2*	*foxl2*	
**AF198086**	***fushi tarazu factor 1b***	***ff1b***	
**AF327373**	***fushi tarazu factor 1c***	***ff1c***	
**AY212920**	***fushi tarazu factor 1d***	***ff1d***	**Testis-enhanced**
**BC066690**	***paired box gene 2a***	***pax2a***	
AF072548	*paired box gene 5*	*pax5*	
**AJ132931**	***presenilin-1***	***pre1***	**Ovary-enhanced**
**BC065382**	***presenilin-2***	***pre2***	**Testis-enhanced**
**BC055549**	***TATA binding protein***	***tbp***	**Ovary-enhanced**
**AF144550**	***Wilms' tumor suppressor gene 1***	***wt1a***	**Testis-enhanced**
**CO359442**	***zebrafish testis-expressed 141***	***zte141***	**Testis-enhanced**
**CO359197**	***zebrafish testis-expressed 15***	***zte15***	**Testis-enhanced**
**CO359335**	***zebrafish testis-expressed 25***	***zte25***	
**CO359148**	***zebrafish testis-expressed 38***	***zte38***	**Testis-enhanced**
**AF331968**	***zona pellucida glycoprotein 2***	***zp2***	**Ovary-enhanced**

* The 18 genes that showed statistically significant differences among the eight organs following a 1-way ANOVA test and a Student-Newman-Keuls post hoc test are displayed in bold. Eleven of these genes that were at least 1.5× more highly expressed in the ovary and/or testis, compared to the common reference, are indicated as ovary- or testis-enhanced. Refer to Table S3 for expression data and references.

Our array data revealed that the candidate gene *zona pellucida glycoprotein 2* (*zp2*; [36]) showed specific expression in the adult zebrafish ovary (81-fold higher expression compared to the common reference target; all subsequent fold values in this publication are in comparison to the common reference target). Zp2 is an egg envelope protein which functions as a secondary sperm receptor during fertilization in mice [37]. Our observations are consistent with previous studies which have also reported ovary-specific expression of *zp2* in zebrafish and mammals [36]–[38].

Another candidate gene, *anti-Müllerian hormone* (*amh*; for reviews see e.g. [39]–[42]), showed a 5.4-fold and 1.2-fold hybridization intensity in the testis and ovary respectively. This is consistent with recent reports indicating higher expression of *amh* in the zebrafish testis compared to the ovary [12], [14], [15], [43]. Similarly, in rat, calf and human, *AMH* is expressed primarily in fetal testicular tissue [44]. Its functions include regression of the Müllerian duct to suppress the formation of the female reproductive tract in males [45], and the regulation of gonadal steroidogenesis to promote testosterone synthesis [46].

Recently, a gene called *fushi tarazu factor 1d (ftz-f1d* or *ff1d)* has been suggested to be a candidate for sex determination and differentiation in zebrafish [12]. *ff1d* is one of the four zebrafish homologs of Steroidogenic Factor-1 (SF-1), a gene implicated in mammalian sex determination and differentiation [12], [47]. Our results revealed that *ff1d* showed an 8.3-fold and 1.4-fold expression level in the testis and ovary respectively. These results are consistent with RT-PCRs performed by [12] which showed that *ff1d* expresses in both gonads of zebrafish, albeit more strongly in the testis.

The positive results obtained from the expression profiles of the 11 candidate genes have enabled us to validate the experimental data obtained from our array, hence reinforcing its reliability. The comparison of candidate gene expression patterns across different organisms will also enable us to broaden our understanding of vertebrate sex determination and differentiation.

### The Gonad Uniclone Microarray reveals many genes with sexually dimorphic expression in the gonads of zebrafish

A 1-way ANOVA parametric test revealed 6302 genes that showed statistically significant differences in their expression levels across the eight organs. These genes were subjected to a Student-Newman-Keuls post hoc test to reveal 1366 and 881 transcripts that showed at least 1.5-fold expression level in the adult ovary (Fig. S2) or testis (Fig. S3) respectively. Such genes will be referred to as genes with ‘ovary-’ or ‘testis-enhanced’ expression. While the expression of some genes appeared to be enhanced in one of the gonads, some showed high expression in both: there were 656 transcripts with levels of at least 1.5-fold in both gonads. A list of selected genes that were highly expressed in the gonads, together with their normalized intensities, is shown in Table 2 (full gene lists with Genbank IDs are shown in Tables S4, S5, S6 under ‘Supporting Information’).

**Table 2:** Selected genes showing >1.5-fold higher expression than the common reference in adult zebrafish gonads (normalized intensities for the gonads, and the range of the normalized intensities for the remaining organs are indicated).

Ovary-expressing genes:				
Gene name	Accession no.	Ovary	Testis	Range for other organs
similar to *egg envelope glycoprotein* isoform 1	CO350790	195.88	0.31	0.35–0.89
similar to *flap structure-specific endonuclease 1*	EV603088	192.56	0.52	0.52–1.35
similar to mKIAA1026 protein	EV561259	154.32	0.38	0.25–4.73
hypothetical protein LOC556628	CO350423	114.75	0.45	0.47–0.99
similar to *low-density lipoprotein receptor dan* isoform 5	EV606521	110.51	3.24	0.51–1.12
*B-cell translocation gene 4*	CO349959	102.79	0.72	0.58–1.07
*zona pellucida glycoprotein 3*	EV560504	99.40	0.21	0.30–1.05
*transcription factor IIIA*	CO349799	86.27	0.97	0.32–0.66
*zona pellucida glycoprotein 2*	AF331968	80.93	0.02	0.03–0.11
*cell division cycle 20* homolog	CO350129	69.31	18.03	0.54–1.23

Testis-expressing genes:				
Gene name	Accession no.	Ovary	Testis	Range for other organs
Unknown	CO353055	0.18	243.77	0.43–1.74
similar to *septin-4* isoform 1	CO353547	0.22	137.44	0.74–1.42
*tektin 1*	CO352798	0.17	127.88	0.40–1.76
similar to mKIAA1148 protein	CO353006	0.09	119.76	0.26–1.64
*dynein,* axonemal, intermediate polypeptide 1	CO355627	0.96	96.42	0.65–1.75
similar to human AKAP-associated sperm protein	CO353327	0.68	80.52	0.68–2.11
*piwi-like 1*	CO354057	7.57	60.42	0.57–1.09
*testis-specific A-kinase-anchoring-protein*	CO354405	1.02	59.27	0.54–1.51
*spermatogenesis associated 4*	CO353245	1.94	57.08	0.48–2.25
*11β–hydroxylase*	EV564930	0.82	13.62	0.76–1.40

Of the 881 ‘testis-enhanced’ genes, 346 showed lower expression levels in the ovary, increasing their differential expression level further between the two gonads. Similarly, 338 of the 1366 ‘ovary-enhanced’ genes showed lower expression level in the testis. Interestingly, many of these genes also appeared to be expressed at a lower level in all the other organs (Fig. S2A). In contrast, the 346 ‘testis-enhanced’ genes showed considerably lower levels in the ovary than in the other organs (Fig. S3C, F and I). Therefore, a higher proportion of genes expressed in the testis, as opposed to those expressed in the ovary, are co-expressed in other organs.

### Genes with the most abundant transcripts in the gonad are expressed in the germline

In order to validate the expression patterns of genes obtained from the microarray, we performed *in situ* hybridizations (ISH) of 16 different transcripts on adult ovary or testis sections. We selected six ovary- and ten testis-enhanced genes on the basis of our microarray data (Table S7).

**Figure 2:**
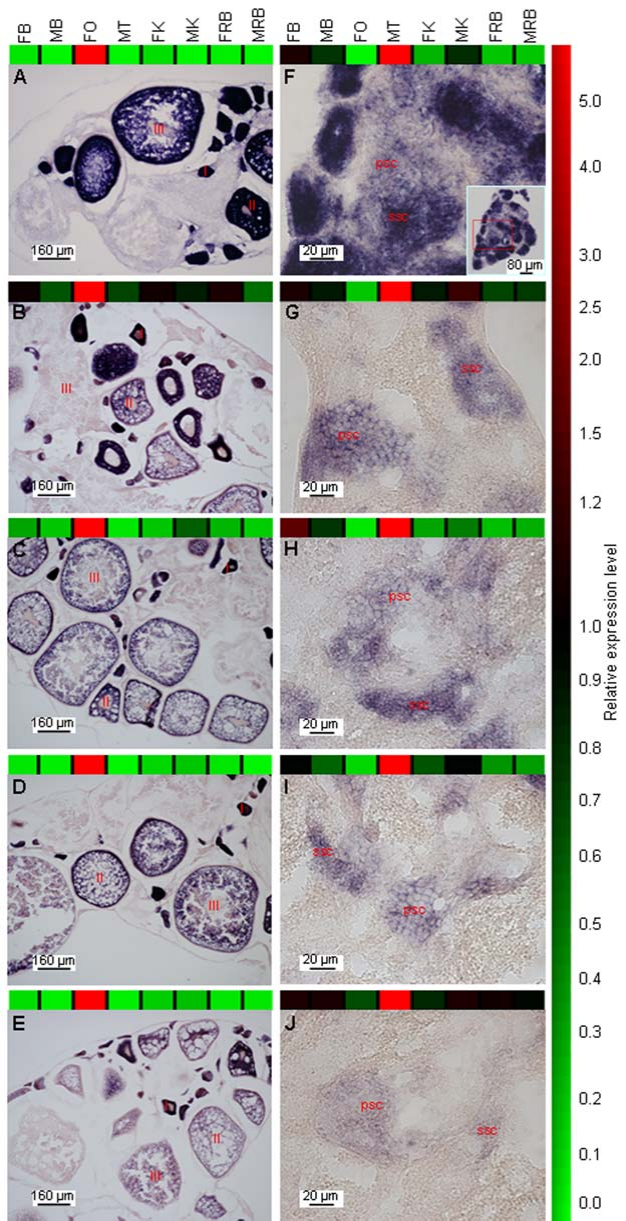
* In situ* hybridization revealed expression in the germline for ovary- and testis-enhanced genes. Adult ovary (A–E) and adult testis (F–J) sections were hybridized with DIG-labeled riboprobes of ten genes which expressed strongly in either one of the gonads. Images are arranged from top to bottom in order of decreasing *in situ* hybridization signal intensity. Microarray expression levels for each gene across the eight organs analyzed are indicated by the coloured expression profiles. (A: *zp2*, B: *btg4*, C: CO350808, D: *gez1*, E: CO350303, F: *gez2*, G: *sept4*, H: CO353006, I: *tekt1*, J: MGC75611; I, II, III: stage I, II and III oocytes; psc: primary spermatocytes, ssc: secondary spermatocytes; FB, FO, FK and FRB represent the brain, ovary, kidney and ‘rest-of-body’ from female zebrafish, while MB, MT, MK and MRB represent the brain, testis, kidney and ‘rest-of-body’ from male zebrafish.)

ISH results showed the expected expression in the gonad for five of the six ovary-expressed genes and eight of the ten testis-expressed genes analyzed (see Table S7), indicating an 81.3% concurrence with our array data. Images of ISH signals in gonad sections for five ovary- and five testis-expressed genes, together with their expression profiles from the microarray, are shown in Fig. 2. Nine of the ten genes shown had a positive correlation of ISH signal intensities with their corresponding gene expression levels as reflected from our array data (Fig. 2; Table S7), hence reinforcing the validity of our hybridization results. Four genes with unknown functions that showed ovary- or testis-enhanced expression were defined in our study as *gez1*-*gez4* (‘*gez’*: *gonad-expressed in zebrafish*; see Fig. 2D, Fig. 2F and Table S7).

All five ovary-enhanced genes with positive signal detected by ISH showed oocyte-specific expression pattern (Fig. 2A–E). Expression was strongest in Stage I and II oocytes, while only some Stage III oocytes showed strong staining. It is likely that expression of these genes is stage-specific and that they are involved in early oogenesis, as shown by their strong expression in earlier stages. Our observation is substantiated by previous studies which showed that *zp2* expression in zebrafish (shown in Fig. 2A) is restricted to developing and not mature oocytes [38], [48]. Similarly, all eight testis-enhanced genes with positive signal detected by ISH showed spermatocyte-specific expression (Fig. 2F–J). Staining was observed in clearly defined clusters of primary and secondary spermatocytes for all genes analyzed. Their expression pattern indicates that these genes may play a role in spermatogenesis.

Most gonadal RNA would be derived from the germline cells since they comprise the most abundant cell types in the gonads. This may explain why all the genes analyzed in our study were found to be expressing in the germline. Analysis of some of the weakly expressed ‘gonad-enhanced’ genes by ISH may in turn reveal genes which are specific to gonadal somatic cells. In addition to validating our array data, our ISH results have also enabled us to identify new gonadal germ cell-markers from zebrafish.

### Microarray data analysis reveals gonad-enhanced expression for several characterized and novel genes

A number of gonad-enhanced genes identified from our array were previously reported to be involved in gonad development and function. For example, the *tektin-1* gene (*tekt1*) and the *11β-hydroxylase* gene (*cyp11b*), which have been implicated in spermatogenesis in mice and humans [49] and testicular differentiation in teleosts [15], [50], showed higher expression levels in the testis (127.8-fold and 13.6-fold respectively).

Some of the gonad-enhanced genes have important molecular functions and could possibly be implicated in gonad development and function. For example, the *cdc20* gene showed 69.3-fold and 18-fold expression levels in the ovary and testis respectively. This WD40-repeat protein, which is a homolog of *fizzy* in *Drosophila* and *Xenopus*, is required to activate the anaphase-promoting complex (APC) to enable anaphase initiation and exit from mitosis [51], [52]. Its strong expression in zebrafish gonads may indicate an involvement in cell division during oogenesis and spermatogenesis.

Of the 2903 gonad-enhanced transcripts, 1008 were found to be novel (without any functional information from zebrafish or other organisms). Studies of these genes with unknown functions can lead to further elucidation of various aspects of zebrafish gonad development and function including gonadal differentiation.

### Several members of the septin gene family are predominantly expressed in the zebrafish gonad

**Figure 3:**
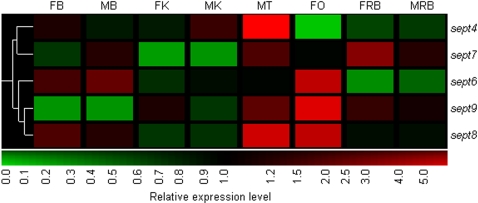
Hierarchical clustering of expression profiles of five members of the *septin* gene family in zebrafish. Expression patterns of *sept6*, *sept9* and *sept8* appeared to be highly correlated to each other while *sept4* and *sept7* formed a separate cluster. *sept4, sept6*, *sept9* and *sept8* were most strongly expressed in either one or both gonads. *sept7* had highest expression in the female ‘rest-of-body’ but strong expression was also observed in the testis. Refer to Fig. 2 for abbreviations.

One member of the *septin* gene family (*septin 4*) is required for normal sperm development in mice [53], [54]. We found that several other members of the *septin* gene family are highly expressed in the gonads of zebrafish (Fig. 3), suggesting a common role of septins in gonad development and/or function. The septins are a family of cytoskeletal GTP-binding proteins found in fungi and animals. They are involved in diverse processes such as cytokinesis, exocytosis, tumorigenesis, maintenance of cell polarity and apoptosis (for reviews, see [55], [56]). Most *septin* transcripts undergo alternative splicing and their splice variants are expressed differentially in various tissues [56], [57].

We identified five members of the *septin* gene family from our array (*sept4, 6, 7, 8* and *9*; Fig. 3). The expression profiles for *sept6*, *sept9* and *sept8* were highly correlated to each other, while those of *sept4* and *sept7* formed a separate cluster. All, except for *sept7*, showed the highest expression levels in either one or both gonads.

Our array data revealed that *sept4*, which has been implicated in various functions from cytokinesis to apoptosis to tumor suppression [58]–[60], was most highly expressed in the testis (137.4-fold), consistently with data from zebrafish [43] and mice [53]. In mice, homozygous *Sept4* mutants have immotile and structurally defective sperm due to a disorganized annulus (a ring-like structure in the sperm tail), indicating a role in spermiogenesis [53], [54]. We performed *in situ* hybridization on adult zebrafish testis sections and found that *sept4* was expressed in the primary and secondary spermatocytes (and possibly round spermatids) (Fig. 2G). This is in contrast to the more restricted expression pattern observed in mouse, where specific testicular localization to post-meiotic round spermatids and spermatozoa was found [53]. Our results, therefore, indicate that *sept4* may be involved in earlier stages of spermatogenesis in zebrafish, possibly in cytokinesis during meiosis.

Another member of the family, *sept9*, showed the highest expression in the gonads, with 4.8-fold and 1.6-fold expression levels in the ovary and testis respectively. Previous reports have shown that *SEPT9* is ubiquitously expressed in human tissues [61], although one isoform shows higher expression in the ovary than testis [62]. Mammalian SEPT9 has been implicated in a wide range of cancers including ovarian cancer [61]–[63].

On the other hand, while *sept7* had the highest expression in the female ‘rest-of-body’, it also showed high expression in the testis (2.3-fold and 1.4-fold expression levels respectively). Both *sept7* and *sept9* have been implicated in germ cell cytokinesis as they localize to germ cell intercellular bridges [64]. Hence, it is possible that these genes may play a role in germ cell cytokinesis in the zebrafish gonad.

Our data also showed that the s*ept6* gene showed the highest expression in the ovary and much lower level in the testis (3.7- vs. 1-fold). High expression levels were also observed in the female and male brains (1.4-fold and 1.8-fold respectively). In contrast, *SEPT6* is ubiquitously expressed in humans with higher levels observed in lymphoid and hematopoietic tissues [57]. It is not known if *sept6* plays a role in gonad development and function but its interaction with *sept9* [65] and its strong expression in the zebrafish ovary indicate a potential involvement.

We have also identified on our array the *sept8* gene which was most highly expressed in the gonads (3.8- and 4.2-fold expression levels in the ovary and testis respectively). Similarly, Blaser and colleagues [66] have shown that one isoform of *SEPT8* was highly expressed in human gonads. While the function of this isoform remains unknown, its strong expression in the gonads may indicate an involvement in gonad development and function. Since Sept8 forms a complex with Sept5 in mouse gonads [66] and since the latter is involved in exocytosis [67], it has been suggested that Sept8 could play a role in the regulation of hormone secretion [66]. In light of the above findings, it would be interesting to further characterize the septins in order to elucidate their potential roles in zebrafish gonad development and/or function.

### The ovarian and testicular transcriptomes of zebrafish are correlated with each other and with those of the male and female brain

**Figure 4:**
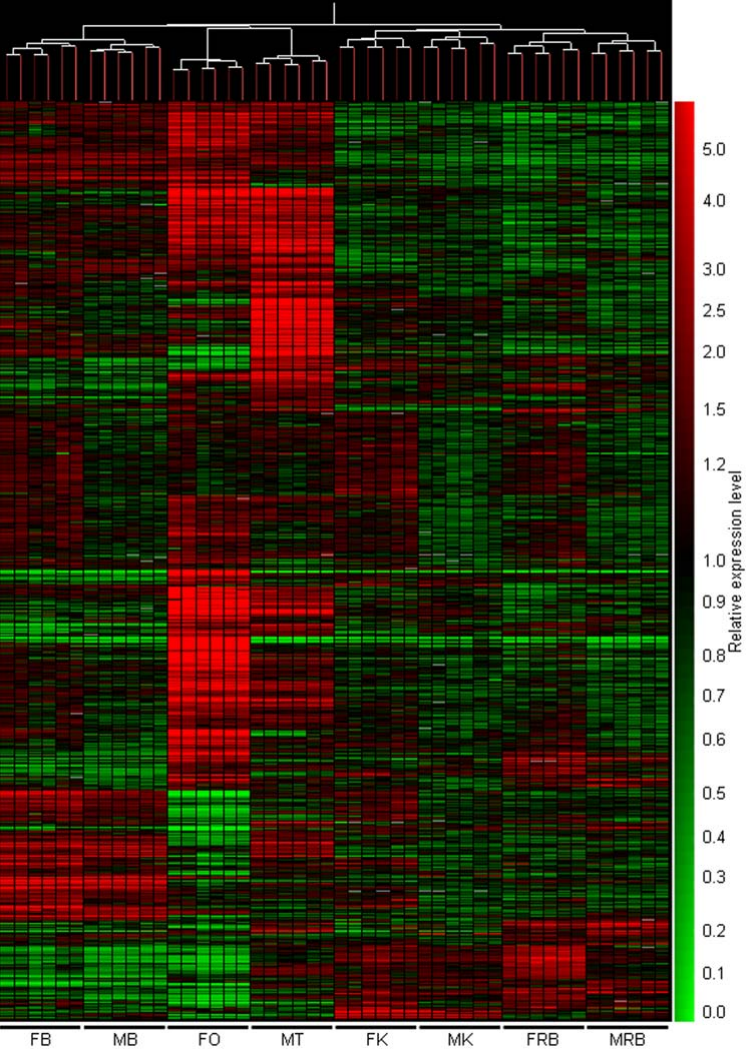
Hierarchical clustering of expression profiles for 6331 genes across eight different organs. Individual gene expression profiles are plotted horizontally against vertical columns of organ type. For each organ, profiles for the three biological replicates and each of their two technical replicates are displayed. Strongly expressed and weakly expressed genes with respect to the common reference are represented in red and green respectively. All organs showed a high level of correlation between biological and technical replicates. The gonads contained the highest number of strongly expressed transcripts. Many genes showed sexually dimorphic gene expression in the gonads and in other organs. Clustering showed correlation between expression profiles of different organs. Kidney targets clustered together with ‘rest-of-body’, while ovary and testis clustered with brain targets to form a distinct group. Refer to Fig. 2 for abbreviations.

In order to compare transcriptional profiles across all eight organs, hierarchical clustering was performed with a similarity measure using Pearson correlation coefficient and a complete linkage algorithm on 6331 genes which had expression levels above those of negative controls. (All negative control probes, *β-actin* probes and probes corresponding to RNA spikes were eliminated from this analysis.) The resulting condition tree (Fig. 4) revealed similar expression levels between biological and technical replicates, highlighting the minimal inter- and intra-array hybridization variation. As expected from an array enriched in gonad-derived clones, the highest number of strongly-expressed transcripts was found in the ovary and testis. Although the organ pairs of the two sexes clustered together, distinct differences were observed for all four sample types tested, particularly between the ovary and testis. In addition, the gonad cluster grouped together with the brain cluster, indicating the similarity of the transcriptome among these organs (Fig. 4). The kidney and ‘rest of body’ profiles showed significantly different transcriptional profiles from those of the gonads and brains.

These data were further substantiated by a principle component analysis (PCA), using a mean centering and scaling, which showed a similar correlation between expression profiles of the different organs, albeit an increased distance was observed between the gonads and brains (Fig. S4). Interestingly, when the ovarian and testis transcriptomes were compared on the PCA, the latter appeared to be more closely related to those of the male and female brain than the former. This further substantiates our finding that a higher proportion of genes in the testis are co-expressed in other organs and previous reports stating the similarity in gene expression of the testis and brain in mice and humans [68], [69]. These data point towards the conservation of gene expression patterns of these organs across different organisms. The discovery of gene mutations in mice and humans which result in defects in both the nervous system as well as the male reproductive system further substantiates the link between brain and testicular gene expression [70], [71].

### Transcriptional analysis reveals genes with sexually dimorphic co-expression in both the gonads and the brains

While searching for genes differentially expressed in the two sexes, we identified 3080 transcripts which showed statistically significant differences in expression between the male and female brain from the 1-way ANOVA parametric test and Student-Newman-Keuls post hoc test. Out of these, 1641 genes were found to be at least 1.5-fold differentially expressed between the brains of the two sexes. When compared to the list of 2247 genes differentially expressed between the two gonads (Tables S4 and S5), we found 276 genes represented in both groups, indicating that their expression was sexually dimorphic between both organ types (Table 3). There were about twice as many genes (183) showing higher expression levels in the female brains than those showing male brain-enhanced expression (93), whereas the number of genes with higher ovary (143) versus higher testis (133) expression was similar (Table 3; for details, see Tables S8 and S9).

**Table 3:** Genes* with sexually dimorphic co-expression in both the gonads and the brains.

	Higher ovary expression	Higher testis expression	Total
Higher FB expression	93	90	183
Higher MB expression	50	43	93
Total	143	133	276

* These genes i) show at least 1.5-fold higher expression than the common reference in one or both gonads as well as in at least one of the two brains; and ii) are differentially expressed (at least 1.5-fold) both between the two gonads and between the two brains.

Sexually dimorphic gene expression has recently been reported in the mouse brain by two research groups [23], [72]. In the first report [23], 54 genes were found to show sexually dimorphic expression in the developing mouse brain (10.5 dpc), with twice as many genes showing enhanced expression in the female brain than in the male brain (36 vs. 18). The second study [72] reported that about 650 genes (14% of those detected from the brain) showed sexually dimorphic expression between the two adult mouse brain types, with about half of them showing higher expression in female brain and the other half in male brain.

**Figure 5:**
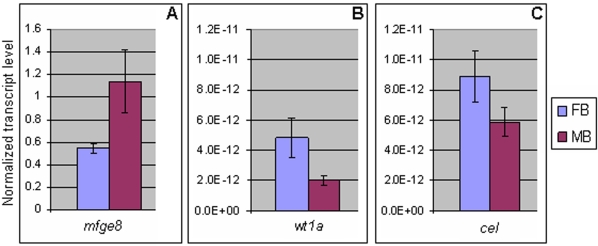
Real-time PCR analysis of selected genes which showed sexually dimorphic expression in the zebrafish brain. Normalized transcript levels in the female (FB) and male brain (MB) for genes *mfge8* (A), w*t1a* (B), and *cel* (C) are shown. The real-time PCR revealed sexually dimorphic expression of these genes in the brain, thereby validating our array data.

We performed real-time PCR on three of the genes showing sexually dimorphic expression in the brain to verify if they were indeed differentially expressed between the male and female adult brain (Fig. 5). These genes included a homolog of the *Xenopus tropicalis* milk fat globule-EGF factor 8 (*mfge8*), Wilms' tumor suppressor gene 1 (w*t1a*) and a homolog of mouse carboxyl ester lipase (*cel*). In all three cases, the real-time PCR results (Fig. 5) yielded differential expression parallel to that observed on the array, thereby confirming our array data. However, only in the case of *mfge8* did we find similar ratios with the two methods; for *wt1a* and *cel*, the difference in expression levels was considerably higher on the array. This may be explained by the fact that brain expression levels of both *wt1a* and *cel* were very low, hence stretching the limit of microarray detection sensitivity.

The three genes analyzed by real-time PCR in our study have previously been implicated in reproduction. In mice, the *Mfge8* gene has been shown to be expressed strongly in the initial segment of the epididymis [73] and has been implicated in cell-cell interactions during fertilization, since the ortholog in pig is expressed on the apical head of the spermatozoa and binds zona pellucida glycoproteins [74], [75]. On the other hand, mutation of *WT1* in humans leads to Wilms' tumor, a pediatric kidney cancer [76], [77]. Multiple organ defects, including in the kidneys and gonads, have been reported in *Wt1* knockout mice [78]. In zebrafish and mice, high expression levels have been observed in the gonads [79]. The *cel* gene is required for conversion of cholesterol esters to free cholesterol for the biosynthesis of steroid hormones. Our observation that these genes are also differentially expressed in the male and female brain indicates their potential involvement in the interplay between the brain and the gonads to regulate gonad development and function.

This is the first report of sexually dimorphic gene expression on a global scale in a teleost brain. The genes we have uncovered that show differences in expression level between the male and female brains and are highly expressed in the gonads could potentially be important for gonad development and function, since it has been shown in birds that a genetically male brain is necessary for normal testis development [16]. Perhaps, the differential expression may be required in the complex regulatory networks involved in the brain-pituitary-gonadal axis that links the nervous and reproductive systems. Such genes are hormonally-influenced and may need to be expressed at different levels in order to establish the differences in the two gonads. Genes differentially expressed in the two brains may also act independently of hormones to directly establish brain sexual differentiation, gender-specific behavior and possibly gonad development and function, as shown recently in birds and mammals [21], [22], [80].

### The Gonad Uniclone Microarray: a useful tool for studies on reproduction in teleosts

The use of the Gonad Uniclone Microarray has enabled us to generate a global profile of gonadal expression patterns in adult zebrafish. We have also been able to identify genes that showed enhanced or sexually dimorphic expression in the gonads. Several genes were also found to be differentially expressed both in the brains and the gonads of the two sexes, suggesting a sexually dimorphic role for these genes in both of these organs. Further analysis of some of these genes may lead to the discovery of gonad- and/or brain-expressed markers with early sex-specificity, provided that their sexually dimorphic expression is also observable at early stages. In addition, the possible availability of the brain for molecular sexing of individuals would enable researchers to preserve the gonad for other studies.

Currently, we are using our array for the analysis of zebrafish gonad differentiation by comparing the expression profiles of developing ovaries, testes and brains isolated from juvenile *vas::egfp*-transgenic individuals sorted on the basis of the dynamic EGFP levels of their gonads [9]. The comparison of the global expression profiles of these developing gonads with each other and with those of the adult organs is expected to provide us with a deeper understanding of the molecular regulation of juvenile hermaphroditism and possibly even that of protogynous sex change.

The Gonad Uniclone Microarray is therefore a useful tool for researchers analyzing the molecular regulation of gonad development and function, and it may also be used to study the involvement of other organs in these processes, since many gonad-enhanced transcripts identified in our study were not exclusively expressed in the gonads. In addition, our array may be suitable for researchers studying genetically-inherited diseases with distinct effects on gonadal phenotype in zebrafish, as well as the genomics of other teleosts for which such a resource is not available [81]. Since several genes have been shown to play similar roles in gonadogenesis in zebrafish and other vertebrates including mammals [13], the data obtained in this study may provide information on the development of the reproductive system in zebrafish, other teleosts and other vertebrates, and may even contribute new pieces to the complex puzzle of the genetic causes of infertility in vertebrates.

## Methods

### Fish stocks and collection of gonadal samples

Zebrafish individuals from the AB strain and from a local strain, called Toh, were kept at our fish facility at ambient temperature and light cycle (12/12 hours) in AHAB (Aquatic Habitats, Apopka, FL, USA) recirculation systems. Adult gonads were isolated as described earlier [34]. For juvenile gonad isolation, the larvae were placed on an examination plate ventral side up, surrounded by 1.5% low melt agarose in egg water (60 µg/ml Instant Ocean sea salts) and overlaid with cold 1× phosphate-buffered saline (PBS). The body cavity was dissected and the gonads were removed using fine forceps (Dumostar #55, Dumont, Switzerland).

### cDNA synthesis and library construction

Normalized and ORESTES cDNA libraries from adult testis were generated earlier [34]. For the generation of three cDNA libraries from differentiating gonads (Table S1), total RNA was isolated from the gonad of 3 and 4 wpf mixed-sex individuals and 5 wpf males. Full-length cDNA was synthesized using Creator Smart Library Construction Kit (Clontech, Mountain View, CA, USA) according to the manufacturer's instruction. After *Sfi I* restriction enzyme digestion, the adaptors and short cDNAs were removed by ChromaSpin 400 column (Clontech). The size fractionated cDNA pool was then cloned into pBS-SK-Sfi (a pBluescript based vector; detailed map is available on request) and transformed into *E. coli* XL10-Gold cells. Clones were sequenced from each library as described earlier; clones from full-length and normalized libraries were sequenced from the 5′ end [34].

For the generation of 4 wpf subtracted libraries, total RNA samples from 4 wpf individuals were sorted into two groups (‘sexed’) using an earlier version of the Gonad Uniclone array (data not shown). Two sets of subtractive hybridizations were performed: 3 wpf gonad (driver) from 4 wpf ‘male gonad’ (tester), and 4 wpf ‘female gonad’ (driver) from 4 wpf ‘male gonad’ (tester). The PCR-Select™ cDNA subtraction kit (Clontech) was used to enrich for developmental stage-specific fragments from the SMART cDNA template according to the recommendations of the manufacturer. To decrease the number of ‘background clones’, mirror-oriented selection was applied as described previously [82].

A 5 wpf transforming male gonad cDNA library was also generated by subtracting against 5 wpf ovary. Zebrafish transgenic to *vas::egfp* were sorted based on their gonadal EGFP levels as transforming males and juvenile females at the age of 5 wpf [9]. cDNA was synthesized from 5 wpf transforming male gonad and 5 wpf ovary total RNA using SMART PCR cDNA synthesis kit (Clontech). PCR-Select™ cDNA subtraction kit (Clontech) was used to enrich for fragments that were present in the 5 wpf transforming male gonad but not in the 5 wpf ovary sample.

The selectively amplified cDNA fragments (in average 400–800 bp in length) were ligated into pGEM-T (Promega, Madison, WI, USA) cloning vector. In total, 1400 clones were picked from the three subtracted libraries and their inserts were sequenced using M13 forward or reverse primer.

### Sequence pre-processing

A total of 4746 ‘raw’ ESTs sequenced from adult testis and ovary, and 11212 ‘raw’ ESTs obtained from differentiating gonad were generated in our laboratory during this study and combined with our previously submitted data set of 11122 ESTs [34]. Pre-processing included the removal of 291 chimeric clones and 86 ESTs containing sequencing errors. In addition, sequences shorter than 100 nucleotides (729 ESTs) were removed together with a set of 1217 ESTs that was repeat-masked over at least 30% of an EST length. The remaining 24813 ESTs were combined with gonad-derived ESTs obtained from Genbank.

Similarly, a total of 90668 gonad-derived ESTs were obtained from GenBank. Chimeric clones (499) and 1568 ESTs that were repeat-masked over at least 30% of an EST length were removed and the remaining 88601 ESTs were added to the 24813 ESTs generated in our laboratory. In addition, a set of 3224 sequences was obtained from NCBI's reference sequence division, thus producing a final data set of 116638 ESTs.

### Clustering and sequence analysis

A total of 116638 ESTs were clustered as described earlier [34]. All ESTs that were <100 nucleotides and masked over at least 30% of an EST length, were reintroduced as singletons after clustering the final data set. We re-introduced those sequences that were included in the Gonad Uniclone macroarray [34] but had been removed during the present pre-processing due to more stringent criteria.

Clones representing transcripts with at least one sequence from our full-length or normalized libraries (i.e. sequences that have not been represented in the previous Gonad UniClone set; [34]) were selected to extend our non-redundant cDNA clone collection (an additional 3634 clones). All the 6370 selected clones were subjected to 3′-end sequencing using an 18mer oligo-dT primer with single-base anchor as described in [83].

Singletons derived from our libraries and clusters without any public ESTs were assessed *in silico* to retain every query EST that does not match data in Genbank since these ESTs represent data that have not been sampled by others. Transcripts were BLAST searched locally against the zebrafish division of Genbank dbEST using default parameters with the exception of e-value <1e-02. BLAST results were filtered by using a sequence similarity threshold defined as 70% sequence identity across 30% of the transcript length. (By using less stringent criteria, we found hits to “weak homologs” and ensured that our new ESTs indeed represent data that had not been sampled in the current public databases.) Sequences that did not show any sequence similarity to zebrafish transcripts were screened against Genbank non-redundant database. The absence of any identifiable homologs in Genbank resulted in the ESTs being classified as either ‘noncoding’ as in the case of sequence identity to intergenic regions or ‘unknown’ in the event that no significant sequence identity was observed (e-value threshold of 1e-02).

### Amplification and purification of cDNA inserts

Adult gonad cDNAs in pBluescript II SK (+) (Stratagene, La Jolla, CA, USA) were amplified in 90 µl colony PCR reactions using the following forward and reverse primers respectively: 5′-GGGCTGCAGGAATTCGGC-3′ and 5′-GGGTTAAGCGGGATATCACTCAG-3′. cDNAs from differentiating gonads in pBS-SK-Sfi were amplified with the following forward and reverse primers respectively: 5′-TCCCAGTCACGACGTTG-3′ and 5′-CCATGATTACGCCAAGC-3′. Control and candidate cDNAs were cloned into pBluescript II SK (+) and pBS-SK-Sfi respectively and were PCR amplified with M13 primers. PCR product quantity and quality were assessed by gel electrophoresis.

A Biomek 2000 workstation (Beckman Coulter, Inc., Fullerton, CA, USA) was used to purify the PCR products by a vacuum-based, size exclusion separation through Montage PCR_μ96_ Plates (Millipore Corporation, Billerica, MA, USA), following the manufacturer's instructions. The DNA was eluted in 50 µl sterile water and separated equally into two 384-well polypropylene plates. The cDNAs were dried completely in a speedvac and then resuspended in 10 µl ArrayIt Micro Spotting Solution Plus (TeleChem International Inc., Sunnyvale, CA, USA). The average concentration of purified PCR products was 180 ng/µl.

### Printing of Gonad Uniclone Microarray

The array was designed according to MIAME guidelines [84]. Purified cDNA inserts were spotted onto amine-coated microarray slides (Genetix, Boston, MA, USA) with 24 Stealth SMP3 Micro Spotting Pins (TeleChem International, Inc., Sunnyvale, CA, USA) using the GeneMachines OmniGrid 100 Microarrayer (Genomic Solutions, Ann Arbor, MI, USA). Spots were printed at an average spot diameter of 145 µm with a vertical and horizontal spot spacing of 180 µm to form a 4×12 subarray layout. A total of 6912 probes were printed in duplicate arrays on each slide. In order to assess spot morphology, red reflect scanning with a 633 nm laser at 10 µm resolution was performed using the Scanarray Express Microarray Scanner (Perkin Elmer, Boston, MA, USA).

The probes comprised 6,370 unique Gonad Uniclones, plus 24 candidate genes in duplicate and several control probes. Positive controls included 132 *β-actin* clones and 111 clones representing 12 other ubiquitously-expressed genes. Negative controls comprised empty cloning vectors (111 pBluescript II SK (+) clones and 18 pBS-SK-Sfi clones), 78 clones representing nine Arabidopsis, viral or fungal genes which showed no sequence similarity to those of zebrafish, triplicates of two clones containing a poly-A tail and flanking vector arms from both cloning vectors which are present in all clones and six samples of spotting solution. Controls were interspersed throughout the array. To validate and monitor target labeling and hybridization, eight Array Control™ PCR Spots (Ambion, Austin, Texas, USA), a set of *E. coli* DNAs designed to hybridize to eight Array Control™ RNA Spikes (Ambion), were included in four concentrations spanning the probe concentration range (25, 60, 120, 250 ng/ul). Information regarding the probes and genes on the Gonad Uniclone Microarray is shown in Table S10 (ArrayExpress accession number: A-MEXP-838). A full list of probes present on the array is listed in Table S11.

### Target amplification and labeling

For generation of labeled targets, five female and five male 10-month-old zebrafish from Toh strain were dissected and total RNA was isolated from the gonad, brain, kidney and ‘rest of body’ using Trizol reagent (Invitrogen, Carlsbad, CA, USA). Following DNase treatment, RNA quantification was performed using the NanoDrop ND-1000 spectrophotometer (NanoDrop Technologies, Inc., Wilmington, DE, USA). RNA quality was assessed by agarose gel electrophoresis and by analyzing RNA samples on the Agilent 2100 Bioanalyzer (Palo Alto, CA, USA) using the RNA 6000 Nano LabChip Kit (Agilent, Santa Clara, CA, USA). The RNA integrity number (RIN) ranged from 6–9 for all samples (a RIN of 1 represents a completely degraded sample whereas a RIN of 10 represents an intact sample). Four of the five individuals with the best RNA quality were selected for subsequent reactions.

Target amplification and labeling were performed using the Amino Allyl MessageAmp™ II aRNA Amplification Kit (Ambion, Austin, TX, USA), following manufacturer's instructions. Total RNA (800 ng) from each target organ was subjected to reverse transcription, second strand synthesis and 1-round amplification by *in vitro* transcription, from which 20 µg amino allyl aRNA was labeled with Alexa Fluor 647 reactive dyes (Invitrogen). For the common reference, 200 ng total RNA from each of the four target organs (gonad, brain, kidney and ‘rest-of-body’) were pooled from one male and one female individual separately and amplified as described above. Ten µg of amino allyl aRNA from each individual was then pooled and labeled with Alexa Fluor 555 reactive dyes. Both aRNA and labeled aRNA were quantified on the NanoDrop ND-1000 spectrophotometer (NanoDrop Technologies, Inc.). Frequency of incorporation (FOI) of the targets ranged from 31–58 dye molecules/1000 nucleotides. Target quality was assessed by agarose gel electrophoresis. Gels of labeled targets were imaged using the Typhoon 9200 Variable Mode Imager and visualized using ImageQuant TL software (GE Healthcare).

A set of eight *E. coli* RNA spikes (10–250 pg) from ArrayControl™ (Ambion) which are complementary to the Array Control PCR Spots on the array were included in each labeling reaction in different ratios as a labeling and hybridization control.

### Hybridization and washing of the microarray

Printed slides were heated on a 100°C heat block for 5 sec after which DNA was UV crosslinked to the slide at 120 mJ using a UV Stratalinker 2400 (Stratagene, La Jolla, CA, USA). The slides were then heated to 100°C for 20 sec after which they were blocked using a protocol adapted from [85]. Slides were immersed and shaken for 1 hr at 40 rpm in a solution of 1.2 g succinic anhydride (Fluka, Buchs, Switzerland) freshly dissolved in 240 ml anhydrous 1,2-dichloroethane (DCE; Fisher Scientific, New Jersey, USA) and 3 ml 1-methylimidazole (Fluka). After blocking, the slides were rinsed once in DCE and three times in sterile water. DNA was denatured by immersing the slides in boiling water for 2 min, after which the slides were dipped twice in 100% ethanol and immediately spin-dried in an Eppendorf 5804 centrifuge.

A total of 32 hybridizations were performed, which included the four pairs of target organs (male and female) from four biological replicates, all hybridized against the common reference. Fifty pmol each of Alexa Fluor 555 and 647 labeled targets were dried completely in a speedvac and dissolved in 50 µl of Micromax hybridization buffer (Perkin Elmer). The targets were denatured at 90°C for 2 min and then incubated at 65°C. A MAUI Mixer FL Hybridization Chamber Lid (BioMicro Systems, Salt Lake City, UT, USA) was adhered onto the slide to form a sealed chamber, allowing 46 µl of target to be loaded onto the array. Hybridization was performed at 65°C for 16 hours on a MAUI 4-Bay Hybridization System (BioMicro Systems) according to the manufacturer's instructions.

The post-hybridization wash protocol was adapted from Diehl et al. [85]. After hybridization, the MAUI Mixer was removed from the slide in a 65°C solution of 2× SSC, 0.1% SDS. The slide was then washed in fresh 2× SSC, 0.1% SDS for 2 min, 1× SSC for 3 min and 0.2× SSC for 3 min. All three washes were performed at room temperature with shaking at 50 rpm. The slides were then dipped into fresh 0.2× SSC and immediately blow-dried using a nitrogen gun at a pressure of 7 bar.

### Microarray scanning and statistical analysis

The arrays were scanned at 5 µm resolution using the ScanArray Express Microarray Scanner. A 543 nm and 633 nm laser with 90% laser power were used to excite Alexa Fluor 555 and 647 fluorophores respectively. Scanned TIFF images of the three biological replicates which gave the best hybridization signal and lowest background were used for statistical analysis (24 images in total). The median of signal and background intensities from the Alexa Fluor 555 and 647 channels were quantified using GenePix Pro 6.0 image acquisition and analysis software (Axon Instruments, Union City, CA, USA).

Statistical analysis was performed using GeneSpring GX 7.3 (Agilent). Local background-subtracted median intensities of 6739 genes were subjected to Locally Weighed Scatterplot Smoothing (Lowess) normalization, global normalization to 33 positive control probes (comprising *β-actin* and nine of the 12 ubiquitously-expressing genes mentioned above) and gene normalization to its median. To eliminate signals that were close to background levels, probes which showed intensities lower than the median raw intensity of 111 negative control probes on each slide were excluded from analysis. (These 111 probes included 99 pBluescript II SK (+) clones, 6 pBS-SK-Sfi clones and 6 clones containing flanking vector arms and poly-A tail.) The resulting probes were subjected to a 1-way ANOVA parametric test (variances assumed equal; p-value cutoff 0.05) and a multiple testing correction using Benjamini and Hochberg False Discovery Rate to reveal probes that showed statistically significant differences in expression across the eight organs. A Student-Newman-Keuls post hoc test was performed on these genes to identify genes that were differentially expressed in a selected organ(s) compared to the others. All target preparation and hybridization protocols as well as raw expression data are available on ArrayExpress (accession no: E-MEXP-1272).

### Quality control of array hybridizations

We adopted the use of a common reference-based design for our experiments. The common reference was generated by pooling equal amounts of targets from the adult gonad, brain, kidney and rest-of-body of both sexes. Such a common reference should hybridize to all spots relevant to the individual target organ hybridizations, thereby enabling a ‘non-zero value’ to be obtained for each spot upon normalization. This strategy has the weakness of being affected by extreme high or low expression level for any given gene in one or more of the organs used.

For each probe, two technical replicates were printed on each microarray. Duplicate spots were located distally from each other to account for any intra-hybridization variation. Scatter plots of background-subtracted raw intensities of Duplicate B against Duplicate A of a single biological replicate of female brain (FB1), male brain (MB2) and ovary (FO3) are shown in Fig. S5. For all three organs, most spots were centered along the 45° line, indicating the similarity in intensities between technical replicates. There were a few spots scattered above or below the line, mostly at the lower signal range. We used the square of the Pearson correlation coefficient (R^2^) as a measure of the relatedness between technical replicates. A high level of correlation was obtained between technical replicates as the median R^2^ value for normalized intensities across all 24 hybridizations was 0.977. These results confirm that the hybridization conditions used for our arrays generated highly reproducible technical replicates, hence reflecting the reliability of our data.

For every organ type, the three best biological replicates (judged on the basis of the linearity of scatter plot) were included in the analysis. Scatter plots of the normalized intensities of various biological replicates of the testis and female brain plotted against each other showed that most genes were centered along the gradient of 1, indicating that the data from the replicates were reproducible (Fig. S6). As with the technical replicates, genes which were scattered above or below the line were mostly at the lower signal range. The biological replicates were confirmed to show highly reproducible signals as a high level of correlation was obtained across all 24 hybridizations (median R^2^ value for normalized intensities = 0.909).

The eight spiked-in controls added in varying ratios during target labeling produced raw/control ratios that were similar to expected ratios (data not shown). This highlights the efficiency and sensitivity of the target labeling, and the reproducibility of target hybridization.

### In situ hybridization

We performed *in situ* hybridization (ISH) for six ovary-expressed and ten testis-expressed genes on adult ovary or testis sections. *Eco RI* or *Spe I*-linearized Gonad Uniclone plasmids containing the cDNAs of interest were *in vitro* transcribed using DIG RNA Labeling Mix (Roche Diagnostics GmbH, Mannheim, Germany) and T7 RNA polymerase (Promega), following manufacturer's instructions. Probes larger than 800 bp were hydrolysed at 60°C using 1× carbonate buffer (40 mM NaHCO_3_, 60 mM Na_2_CO_3_) to generate approximately 300 bp fragments.

For ISH, we used a modified protocol based on Strahle et al. [86]. Dissected gonads from two female and two male adult zebrafish (AB strain) were fixed overnight at 4°C in 4% paraformaldehyde in 1× PBS. Ovary and testis sections (20 µm and 18 µm respectively) were made using a cryostat (Leica Microsystems GmbH, Nussloch, Germany), transferred to microscope slides and hybridized overnight at 70°C with DIG-labeled riboprobes at a final concentration of 1 ng/µl. Slides were blocked in a solution containing MABT (5× maleic acid buffer, 20% Tween 20, pH 7.5), 20% fetal bovine serum and 2% blocking reagent (Roche) for 2 hours at room temperature, followed by overnight incubation in a 1:3000 dilution of anti-digoxigenin-AP antibody (Roche) in blocking solution. Slides were stained overnight at room temperature in a solution of NBT (337.5 µg/ml) and BCIP (175 µg/ml) (Roche) in AP staining buffer (100 mM NaCl, 50 mM MgCl_2_, 100 mM Tris pH 9.5, 0.1% Tween-20). Slides were viewed under a Zeiss Axioplan 2 upright microscope and imaged using a Nikon DXM1200F digital camera and ACT-1 software.

### Real-time PCR

Real-time PCRs were performed on three genes that showed differential expression between the male and female brain in the microarray data. Total brain RNA (300 ng) from three male and three female adult individuals was reverse transcribed using the iScript cDNA Synthesis Kit (Bio-Rad Laboratories, Inc., Hercules, CA, USA) following manufacturer's instructions. The RNA used was identical to that used as targets for array hybridization. cDNA (0.5–1 µl) was amplified in 25 µl triplicate reactions containing 0.2 µM primers and 12.5 µl iQ SYBR Green Supermix (Bio-Rad) using a MyiQ Single Colour Real-time PCR Detection System (Bio-Rad), according to manufacturer's instructions. Primers used for amplification were as follows: *mfge8*: F-5′ TTATCAAGGCTTTCAAGGTGGC 3′, R-5′ GGCTTTACGGCAGACAACAGG 3′, w*t1a*: F-5′ GAGCCATCCCGGAGGTTATGA 3′, R-5′ TTGGTCTCGGTTGAACGCACA 3′; *cel*: F-5′ ACATTCCCTCAATCAACAACGC 3′, R-5′ CAAAGGCTTTCCAAACACATACTG 3′;. Either a plasmid DNA or cDNA dilution series was used to generate a standard curve for quantification of transcript levels and for measuring amplification efficiency. For each sample, *β–actin* was amplified in parallel using the following primers: F-5′ CCATCCTTCTTGGGTATGGAATC 3′, R-5′ GGTGGGGCAATGATCTTGATC 3′, to normalize transcript levels. Negative control PCRs containing RNA templates and *β–actin* primers were included for each individual to rule out the possibility of genomic DNA contamination.

## Supporting Information

Figure S1 Expression profiles of selected candidate genes across eight organs in adult zebrafish. The normalized intensity (in log scale) is indicated on the y-axis and organ types are stated on the x-axis. FB, FO, FK and FRB represent the brain, ovary, kidney and ‘rest-of-body’ from female zebrafish, while MB, MT, MK and MRB represent the brain, testis, kidney and ‘rest-of-body’ from male zebrafish.
(1.28 MB TIF)

Figure S2 Self-organizing map showing the 1366 genes which had at least 1.5-fold higher expression in the ovary compared to the common reference target. The genes were clustered using 1000000 iterations and a neighbourhood radius of 3.0. The number of genes in each cluster is indicated in parentheses. FB, FO, FK and FRB represent the brain, ovary, kidney and ‘rest-of-body’ from female zebrafish, while MB, MT, MK and MRB represent the brain, testis, kidney and ‘rest-of-body’ from male zebrafish.
(4.58 MB TIF)

Figure S3 Self-organizing map showing the 881 genes which had at least 1.5-fold higher expression in the testis compared to the common reference target. The genes were clustered using 1000000 iterations and a neighbourhood radius of 3.0. The number of genes in each cluster is indicated in parentheses. FB, FO, FK and FRB represent the brain, ovary, kidney and ‘rest-of-body’ from female zebrafish, while MB, MT, MK and MRB represent the brain, testis, kidney and ‘rest-of-body’ from male zebrafish.
(4.69 MB TIF)

Figure S4 Principle component analysis showing correlation between expression profiles of eight different organs in the three biological replicates analyzed. Kidney targets clustered together with ‘rest of body’ to form a distinct group from the ovary, testis and brain targets. The brain targets also showed a higher correlation to the testis than the ovary. FB, FO, FK and FRB represent the brain, ovary, kidney and ‘rest-of-body’ from female zebrafish, while MB, MT, MK and MRB represent the brain, testis, kidney and ‘rest-of-body’ from male zebrafish.
(1.75 MB TIF)

Figure S5 Typical scatter plots showing reproducibility of technical replicates in the following hybridizations: A) female brain (FB), B) male brain (MB) and C) ovary (FO). Background-subtracted raw intensities of Replicate B against Replicate A of a single biological replicate are shown. All hybridizations were performed against a common reference sample containing an equal mixture of labeled targets from adult gonads, brains, kidney and ‘rest-of-body’ from the two sexes.
(2.47 MB TIF)

Figure S6 Scatter plots showing reproducibility of the three biological replicates for testis (MT) and female brain (FB) hybridizations. The normalized intensities of various biological replicates are plotted against each other. A: MT2 vs MT1, B: MT3 vs MT1, C: MT3 vs MT2, D: FB2 vs FB1, E: FB3 vs FB1, F: FB3 vs FB2.
(2.30 MB TIF)

Table S1 Description of the gonad-derived libraries and ESTs used for the extension of the dataset featured on the Gonad Uniclone Microarray. (Abbreviations: FL-full-length, NRM-normalized, ORE-ORESTES, R-random, S/SU-subtracted, wpf-week post fertilization)
(0.02 MB XLS)

Table S2 Domains and corresponding functional classes of 24 clones which did not find hits to characterized genes in Genbank when BLAST-searched at the nucleotide level. *Source: ‘non-coding’: transcripts align to non-coding regions of the zebrafish genome; ‘no hits’: transcripts do not find any significant matches to the zebrafish genome.
(0.02 MB XLS)

Table S3 Normalized intensities (‘Norm’) and corresponding standard error (‘StdErr Norm’) of the 24 candidate genes on the Gonad Uniclone Microarray. Genes which were at least 1.5× more highly expressed in the ovary or testis, compared to the common reference, are underlined or indicated in bold respectively. References which describe the characterization of these genes are also indicated. (FB, FO, FK, FRB: brain, ovary, kidney, ‘rest-of-body’ from female zebrafish; MB, MT, MK, MRB: brain, testis, kidney, ‘rest-of-body’ from male zebrafish. Data from 3 biological replicates is shown.)
(0.09 MB XLS)

Table S4 List of 1366 genes with at least 1.5-fold higher expression in the adult ovary compared to the common reference. Normalized intensities (‘Norm’) and corresponding standard errors (‘StdErr Norm’) are shown. (FB, FO, FK, FRB: brain, ovary, kidney, ‘rest-of-body’ from female zebrafish; MB, MT, MK, MRB: brain, testis, kidney, ‘rest-of-body’ from male zebrafish.)
(0.63 MB XLS)

Table S5 List of 881 genes with at least 1.5-fold higher expression in the adult testis compared to the common reference. Normalized intensities (‘Norm’) and corresponding standard errors (‘StdErr Norm’) are shown. (FB, FO, FK, FRB: brain, ovary, kidney, ‘rest-of-body’ from female zebrafish; MB, MT, MK, MRB: brain, testis, kidney, ‘rest-of-body’ from male zebrafish.)
(0.41 MB XLS)

Table S6 List of 656 genes with at least 1.5-fold higher expression in both the adult ovary and testis compared to the common reference. Normalized intensities (‘Norm’) and corresponding standard errors (‘StdErr Norm’) are shown. (FB, FO, FK, FRB: brain, ovary, kidney, ‘rest-of-body’ from female zebrafish; MB, MT, MK, MRB: brain, testis, kidney, ‘rest-of-body’ from male zebrafish.)
(0.32 MB XLS)

Table S7 List of highly-expressed ovary and testis genes analyzed by *in situ* hybridization (ISH). Normalized intensities for ovary (FO) and testis (MT) targets and ratios of normalized intensities of FO/MT (or vice versa) are shown.
(0.02 MB XLS)

Table S8 List of 183 gonad-expressed genes that show female brain-enhanced expression. These genes i)show at least 1.5-fold higher expression than the common reference in the female brain and one or both gonads; and ii) are differentially expressed (at least 1.5-fold) both between the gonads and between the brains of the two sexes. Normalized intensities (‘Norm’) and corresponding standard errors (‘StdErr Norm’) are shown, along with the ratios of normalized intensities of female brain:male brain (‘Ratio FB/MB’) and ovary:testis (‘Ratio FO/MT’). (FB, FO, FK, FRB: brain, ovary, kidney, ‘rest-of-body’ from female zebrafish; MB, MT, MK, MRB: brain, testis, kidney, ‘rest-of-body’ from male zebrafish.)
(0.11 MB XLS)

Table S9 List of 93 gonad-expressed genes that show male brain-enhanced expression. These genes i)show at least 1.5-fold higher expression than the common reference in the male brain and one or both gonads; and ii) are differentially expressed (at least 1.5-fold) both between the gonads and between the brains of the two sexes. Normalized intensities (‘Norm’) and corresponding standard errors (‘StdErr Norm’) are shown, along with the ratios of normalized intensities of male brain:female brain (‘Ratio MB/FB’) and ovary:testis (‘Ratio FO/MT’). (FB, FO, FK, FRB: brain, ovary, kidney, ‘rest-of-body’ from female zebrafish; MB, MT, MK, MRB: brain, testis, kidney, ‘rest-of-body’ from male zebrafish.)
(0.07 MB XLS)

Table S10 Composition of probes and genes on Gonad Uniclone Microarray.
(0.01 MB XLS)

Table S11 Complete list of probes on Gonad Uniclone Microarray
(1.10 MB XLS)
