# Supplementary figures and images for: Transcriptomic Analyses Reveal Novel Genes with Sexually Dimorphic Expression in the Zebrafish Gonad and Brain
Source: PLoS One. 2008 Mar 12;3(3):e1791. doi: 10.1371/journal.pone.0001791 (PMC2262149; doi:10.1371/journal.pone.0001791)

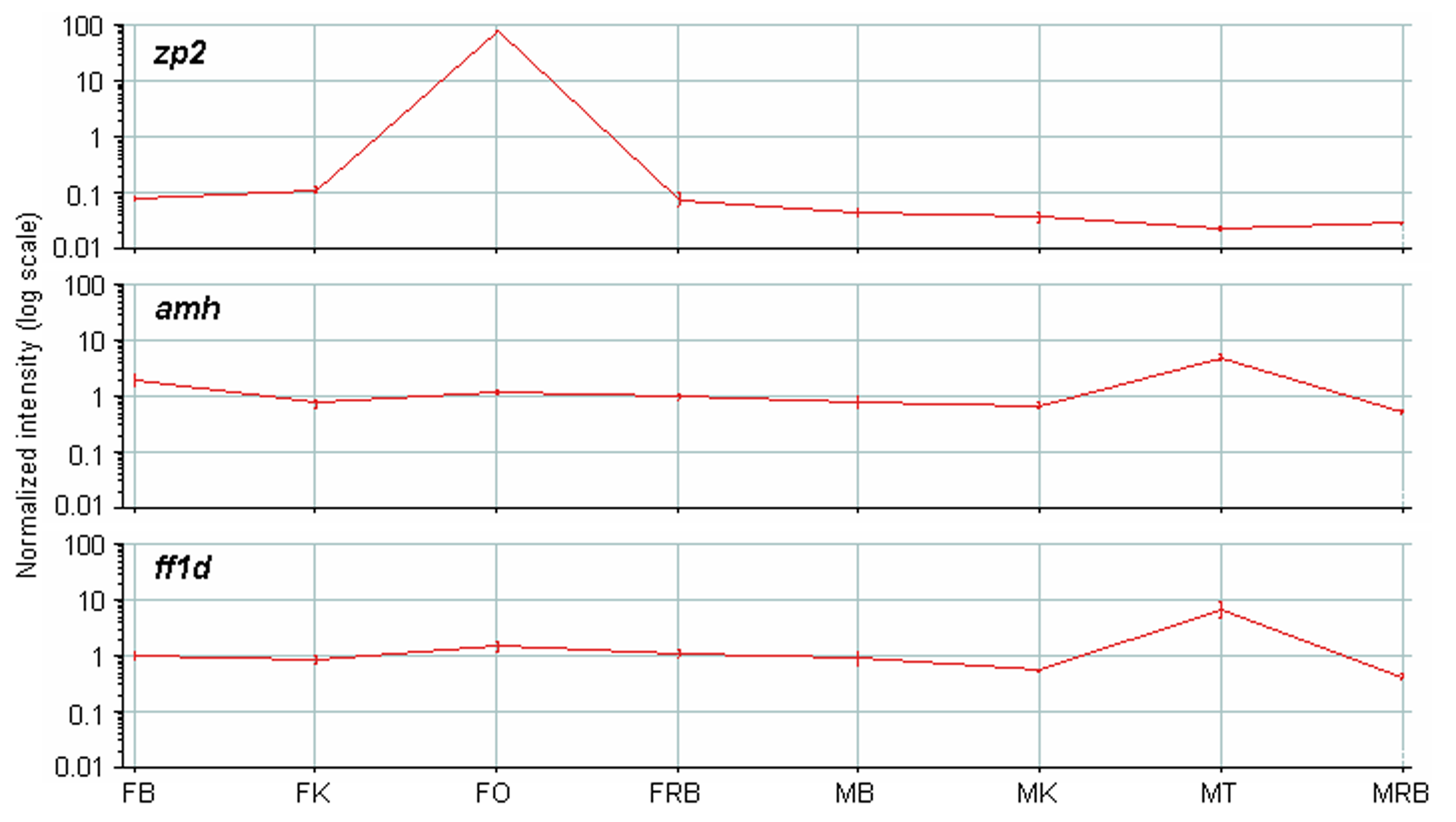

Supplement: Figure S1 — Expression profiles of selected candidate genes across eight organs in adult zebrafish. The normalized intensity (in log scale) is indicated on the y-axis and organ types are stated on the x-axis. FB, FO, FK and FRB represent the brain, ovary, kidney and ‘rest-of-body’ from female zebrafish, while MB, MT, MK and MRB represent the brain, testis, kidney and ‘rest-of-body’ from male zebrafish. (1.28 MB TIF) [file pone.0001791.s001.tif]

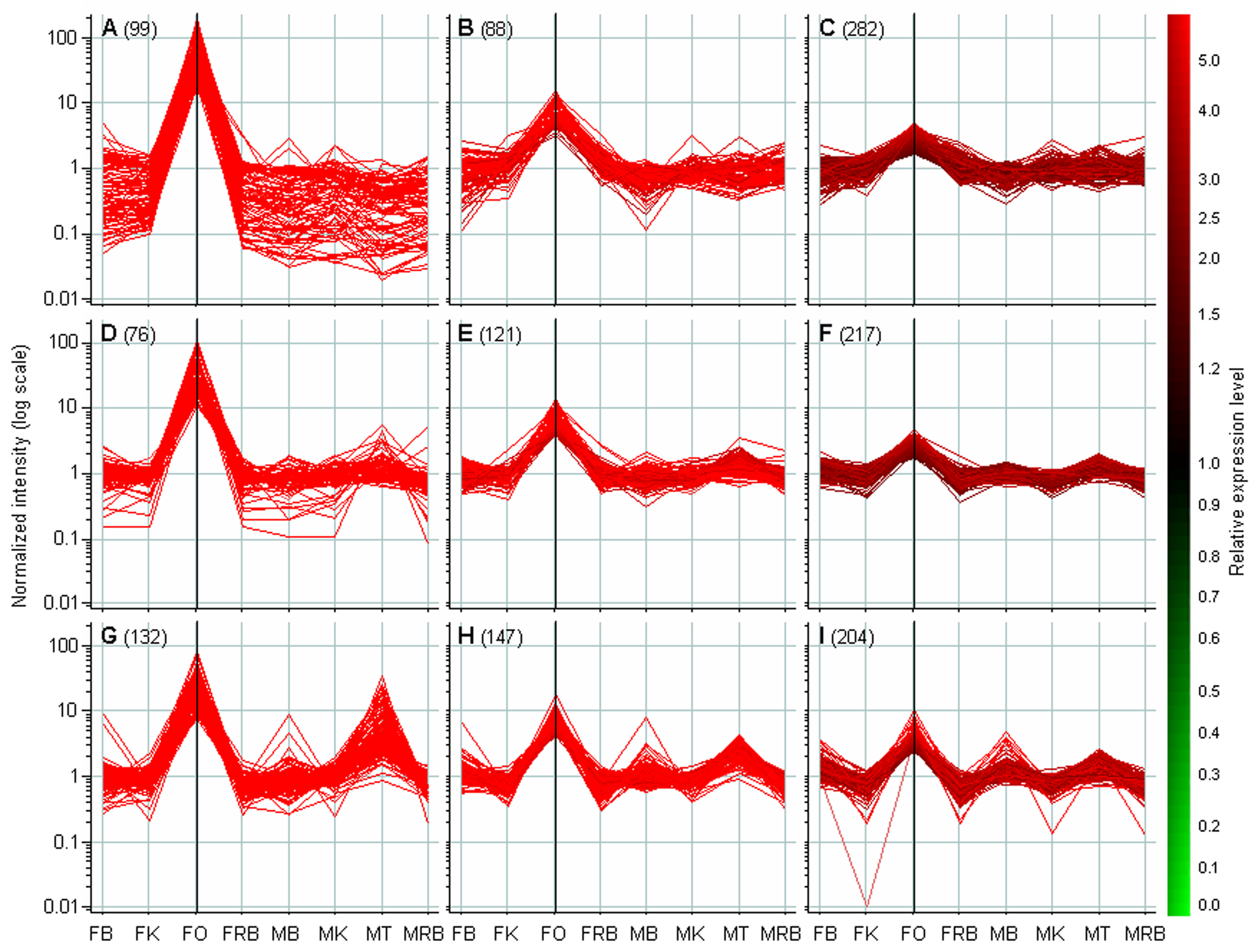

Supplement: Figure S2 — Self-organizing map showing the 1366 genes which had at least 1.5-fold higher expression in the ovary compared to the common reference target. The genes were clustered using 1000000 iterations and a neighbourhood radius of 3.0. The number of genes in each cluster is indicated in parentheses. FB, FO, FK and FRB represent the brain, ovary, kidney and ‘rest-of-body’ from female zebrafish, while MB, MT, MK and MRB represent the brain, testis, kidney and ‘rest-of-body’ from male zebrafish. (4.58 MB TIF) [file pone.0001791.s002.tif]

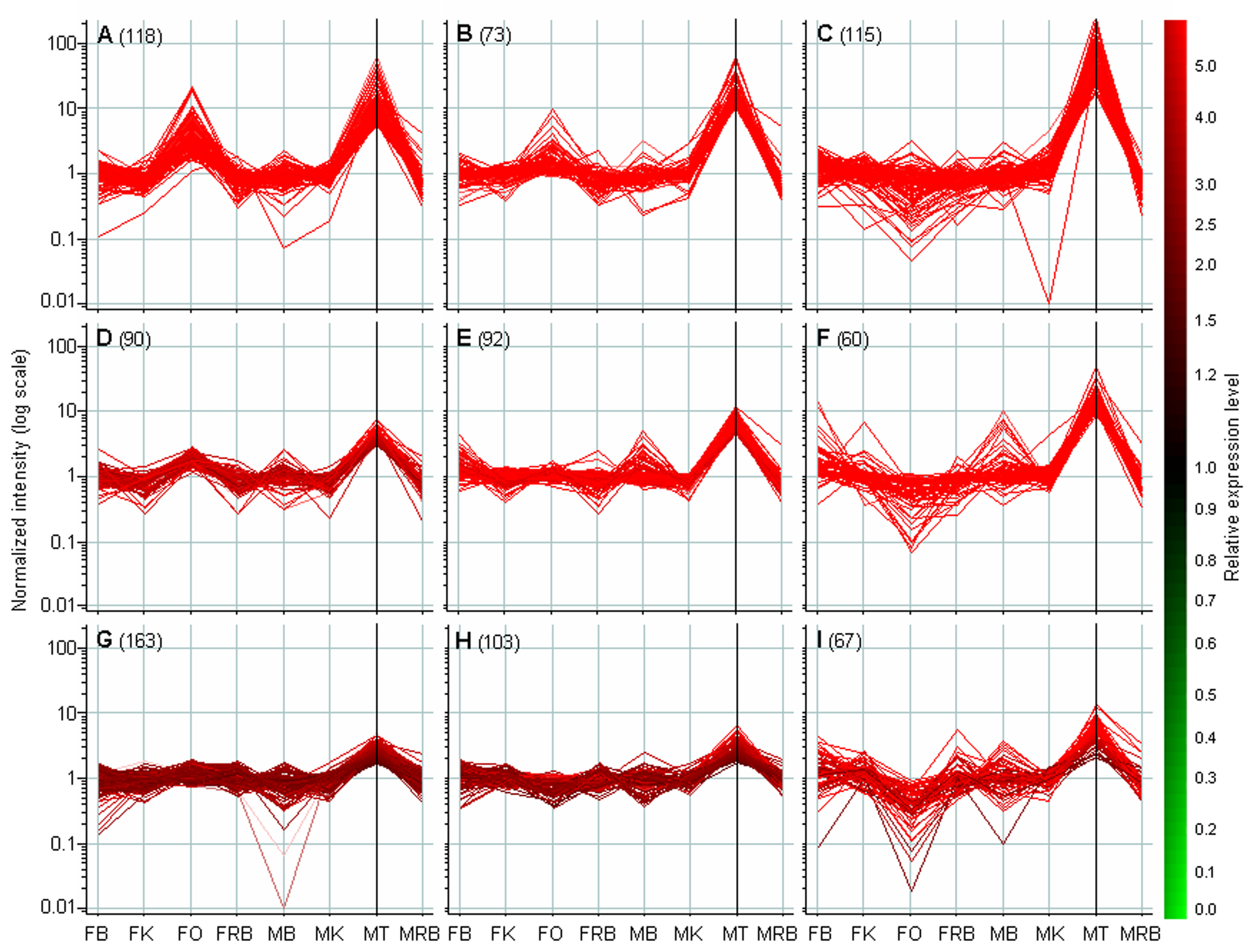

Supplement: Figure S3 — Self-organizing map showing the 881 genes which had at least 1.5-fold higher expression in the testis compared to the common reference target. The genes were clustered using 1000000 iterations and a neighbourhood radius of 3.0. The number of genes in each cluster is indicated in parentheses. FB, FO, FK and FRB represent the brain, ovary, kidney and ‘rest-of-body’ from female zebrafish, while MB, MT, MK and MRB represent the brain, testis, kidney and ‘rest-of-body’ from male zebrafish. (4.69 MB TIF) [file pone.0001791.s003.tif]

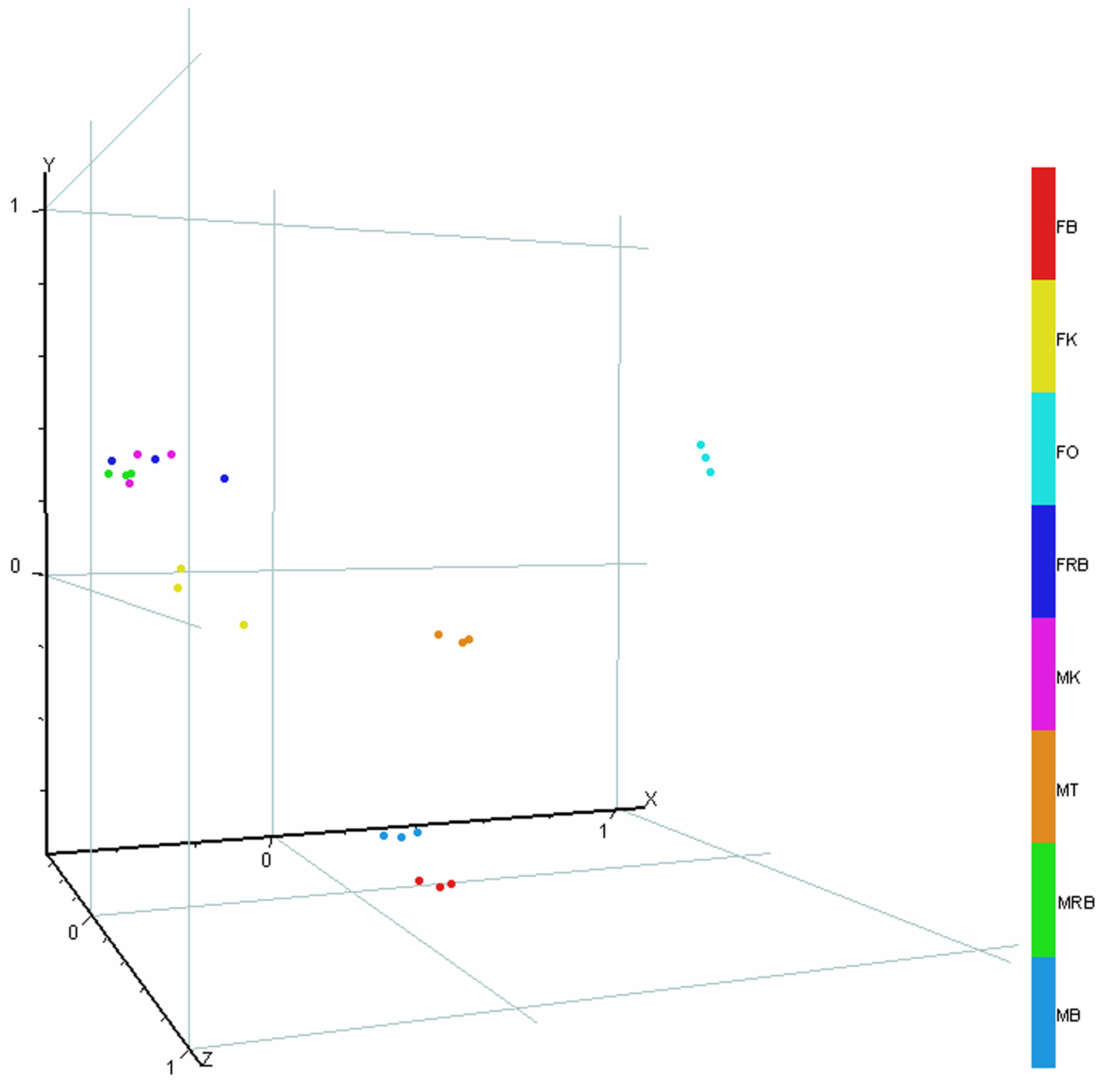

Supplement: Figure S4 — Principle component analysis showing correlation between expression profiles of eight different organs in the three biological replicates analyzed. Kidney targets clustered together with ‘rest of body’ to form a distinct group from the ovary, testis and brain targets. The brain targets also showed a higher correlation to the testis than the ovary. FB, FO, FK and FRB represent the brain, ovary, kidney and ‘rest-of-body’ from female zebrafish, while MB, MT, MK and MRB represent the brain, testis, kidney and ‘rest-of-body’ from male zebrafish. (1.75 MB TIF) [file pone.0001791.s004.tif]

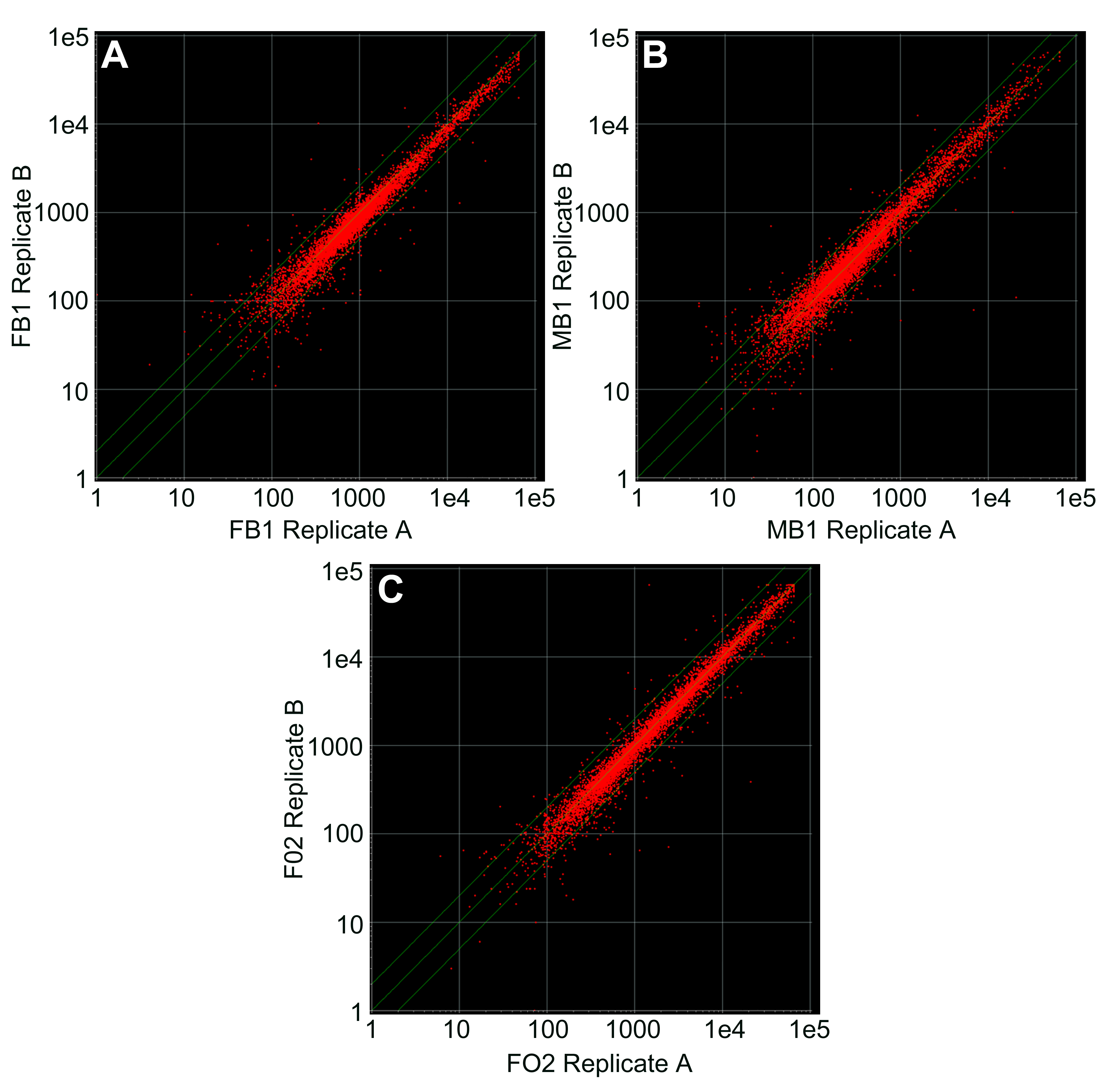

Supplement: Figure S5 — Typical scatter plots showing reproducibility of technical replicates in the following hybridizations: A) female brain (FB), B) male brain (MB) and C) ovary (FO). Background-subtracted raw intensities of Replicate B against Replicate A of a single biological replicate are shown. All hybridizations were performed against a common reference sample containing an equal mixture of labeled targets from adult gonads, brains, kidney and ‘rest-of-body’ from the two sexes. (2.47 MB TIF) [file pone.0001791.s005.tif]

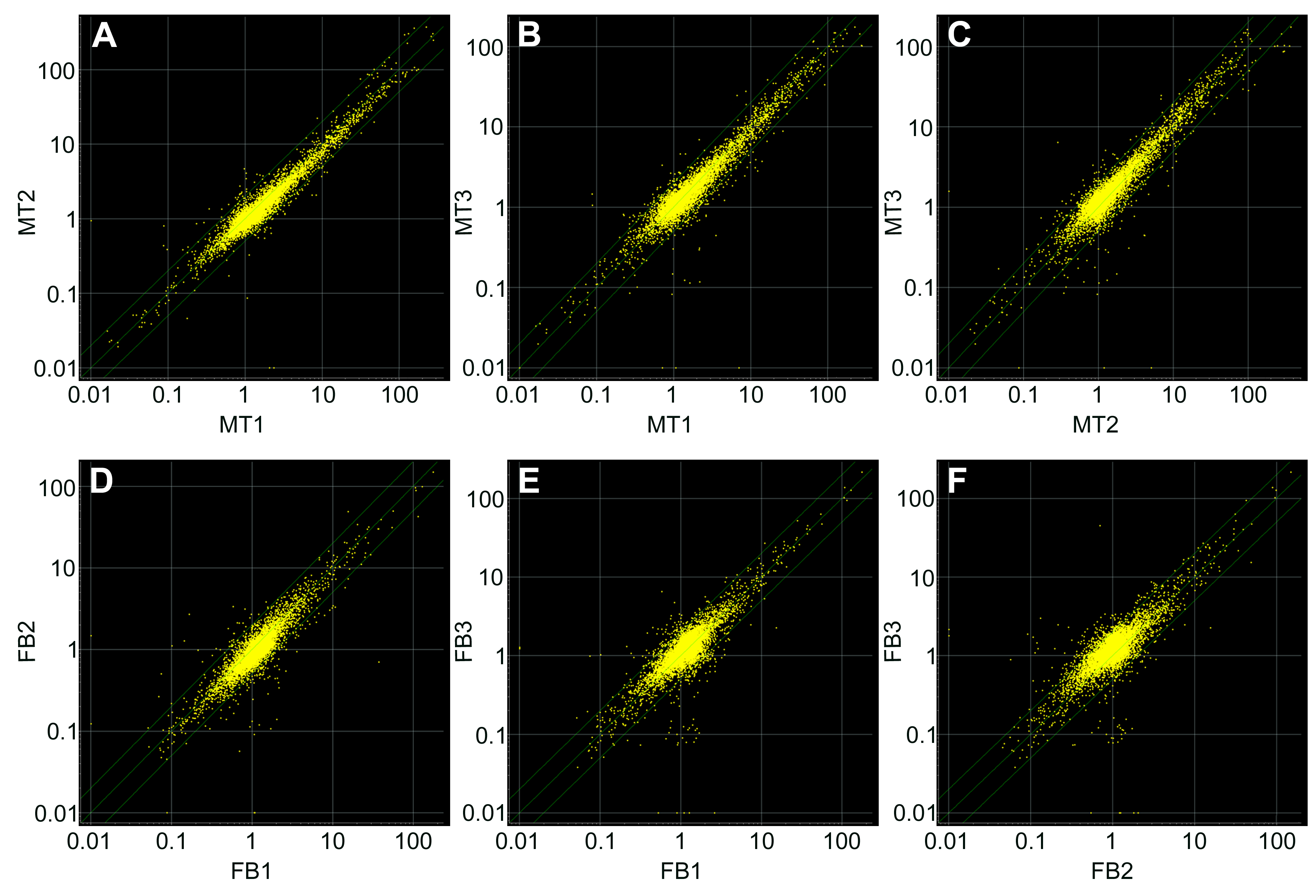

Supplement: Figure S6 — Scatter plots showing reproducibility of the three biological replicates for testis (MT) and female brain (FB) hybridizations. The normalized intensities of various biological replicates are plotted against each other. A: MT2 vs MT1, B: MT3 vs MT1, C: MT3 vs MT2, D: FB2 vs FB1, E: FB3 vs FB1, F: FB3 vs FB2. (2.30 MB TIF) [file pone.0001791.s006.tif]
